# New system to examine the activity and water and food intake of germ-free mice in a sealed positive-pressure cage

**DOI:** 10.1016/j.heliyon.2019.e02176

**Published:** 2019-08-14

**Authors:** Kimie Niimi, Eiki Takahashi

**Affiliations:** Research Resources Division, RIKEN Center for Brain Science, 2-1 Hirosawa, Wako-shi, Saitama 351-0198, Japan

**Keywords:** Microbiology, Neuroscience, Physiology

## Abstract

Germ-free (GF) mice are useful models for the examination of host–microbe interactions in health and disease. We recently reported on the maintenance of individual GF mice for more than 1 year in a sealed positive-pressure cage. However, no useful system exists to automatically record basic behavioral patterns, such as activity and the intake of water and food, under GF status. In this study, we examined basic behavior by combining the sealed positive-pressure cage with a behavioral monitoring system and observed the gross morphology of GF mice at 4 weeks and 8 months of age. GF mice exhibited cecal enlargement and had lower body and adipose tissue weights compared with age-matched specific pathogen–free (SPF) mice. Although both strains had similar circadian rhythms, GF mice exhibited decreased activity compared with age-matched SPF mice. GF mice also exhibited increased levels of water intake compared with age-matched SPF mice. Although GF mice demonstrated decreased food intake levels at the age of 4 weeks, they exhibited increased food intake levels compared with age-matched SPF mice at the age of 8 months. The present research indicates that automated measurement systems that record the basic behaviors of GF mice for long periods are useful for the acceleration of the study of metabolic functions and host–microbe interactions.

## 1 Introduction

Intestinal commensal bacteria play important roles in the health and disease states of their host organisms by participating in nutrient metabolism and influencing immune responses [1]. Recent studies have documented strong associations between specific intestinal microbiota and several diseases, such as obesity [2, 3, 4] and metabolic [5, 6], autoimmune [3, 7], and central nervous system [8, 9] diseases, as well as bone homeostasis [10]. Gnotobiotic mice have been used for research related to the microbiome, and are established by inoculating germ-free (GF) mice with one or more strains of microorganisms. The organs of GF mice provide empty niches that can be populated with different microbiota, including those from human patients, which provides the opportunity to determine the causative roles of certain bacterial communities for specific diseases.

Gnotobiotic animals are usually raised and maintained using GF techniques under isolator conditions to prevent microbial contamination. GF mice are generally maintained in flexible film isolators designed to preserve the sterility of their environment. To house and maintain animals under GF conditions, the introduction and removal of supplies and samples to and from the isolator requires a time-consuming and labor-intensive decontamination process that is conducted before and after the port to the isolator is opened. Colonization experiments that use different microbial conditions require multiple isolators to prevent cross-contamination, necessitating a large space. Alternative caging systems that allow better accessibility while maintaining sterility and avoiding contamination would be useful to reduce the complexity and difficulty of working with these animals. To overcome these disadvantages, the use of sealed positive-pressure (SPP) cages has recently been reported. The successful maintenance of multiple groups of gnotobiotic mice for 2 weeks was achieved [11]. Another group reported the successful rearing of GF mice for 12 weeks in a positive-pressure Isocage (ISOcage P; Tecniplast SpA, Buguggiate, Varese, Italy) [12]. Additionally, we recently reported the successful maintenance of individual GF mice for more than 1 year in the Sentry SPP isolation cage (Allentown, Inc., Allentown, NJ, USA) [13].

Items such as mice and measuring equipment can be difficult for an individual to handle in an isolator while wearing isolator gloves. Although SPP cages are advantageous for the maintenance and breeding of GF mice, they have not been reported to allow for the careful analysis of the behavior of these mice.

In this study, we developed a novel behavioral testing system that features the automated collection of data on GF mice, such as activity and the intake of food and water, and we provide experimental data demonstrating the use of this system with GF mice. Our basic behavioral recording platform provides a prototype for use in the next generation of GF mouse studies.

## 2 Materials and methods

### 2.1 Animals

This study was approved and overseen by the Animal Experiments Committee of RIKEN (Saitama, Japan), and was conducted in accordance with the Institutional Guidelines for Experiments using Animals. Three-week-old GF and specific pathogen–free (SPF) mice with the C57BL/6NJcl genetic background were delivered from CLEA Japan, Inc. (Tokyo, Japan). In our facility, SPF status means that the mouse is free of the following microorganisms: mouse hepatitis virus, Sendai virus, Ectromelia virus, lymphocytic choriomeningitis virus, mouse rotavirus, mouse parvovirus, mouse encephalomyelitis virus, pneumonia virus of mice, mouse adenovirus, reovirus type 3, lactate dehydrogenase elevating virus, *Mycoplasma pulmonis*, *Salmonella typhimurium*, *Clostridium piliforme*, *Corynebacterium kutscheri*, *Pasteurella pneumotropica*, cilia-associated respiratory bacillus, *Escherichia coli* O115a, *Helicobacter hepaticus*, *Pseudomonas aeruginosa*, *Pneumocystis carinii*, *Syphacia obvelata*, and *Aspiculuris tetraptera*.

GF and SPF mice were housed individually in the Sentry SPP isolation cage until use. The mice were reared according to our previously described procedures [13]. Microbiological tests to confirm the GF or SPF status were conducted by the ICLAS Monitoring Center (Kanagawa, Japan). To examine the GF status, fresh fecal samples, soiled bedding, swabs from cages, and RO water from the drinking bottles were collected and microbiologically tested using previously reported methods [13]. In the microbiological tests, cooked meat broth and thioglycollate medium were used for anaerobic bacterial culture, whereas heart infusion broth was used for aerobic culture. All bacterial cultures were incubated at 37 °C or room temperature for 14 days. For fungal culture, potato dextrose broth inoculated with the samples was incubated at room temperature for 14 days.

Separate groups of mice were used for gross morphological experiments (including body weight and adipose tissue weight measurements) at the ages of 4 weeks (10 GF mice, 8 SPF mice) and 8 months (10 GF mice, 10 SPF mice), activity and water intake tests at the ages of 4 weeks (10 GF mice, 8 SPF mice) and 8 months (10 GF mice, 10 SPF mice), and food intake tests at the ages of 4 weeks (9 GF mice, 8 SPF mice) and 8 months (10 GF mice, 9 SPF mice).

### 2.2 Behavioral experiments

**Fig. 1:**
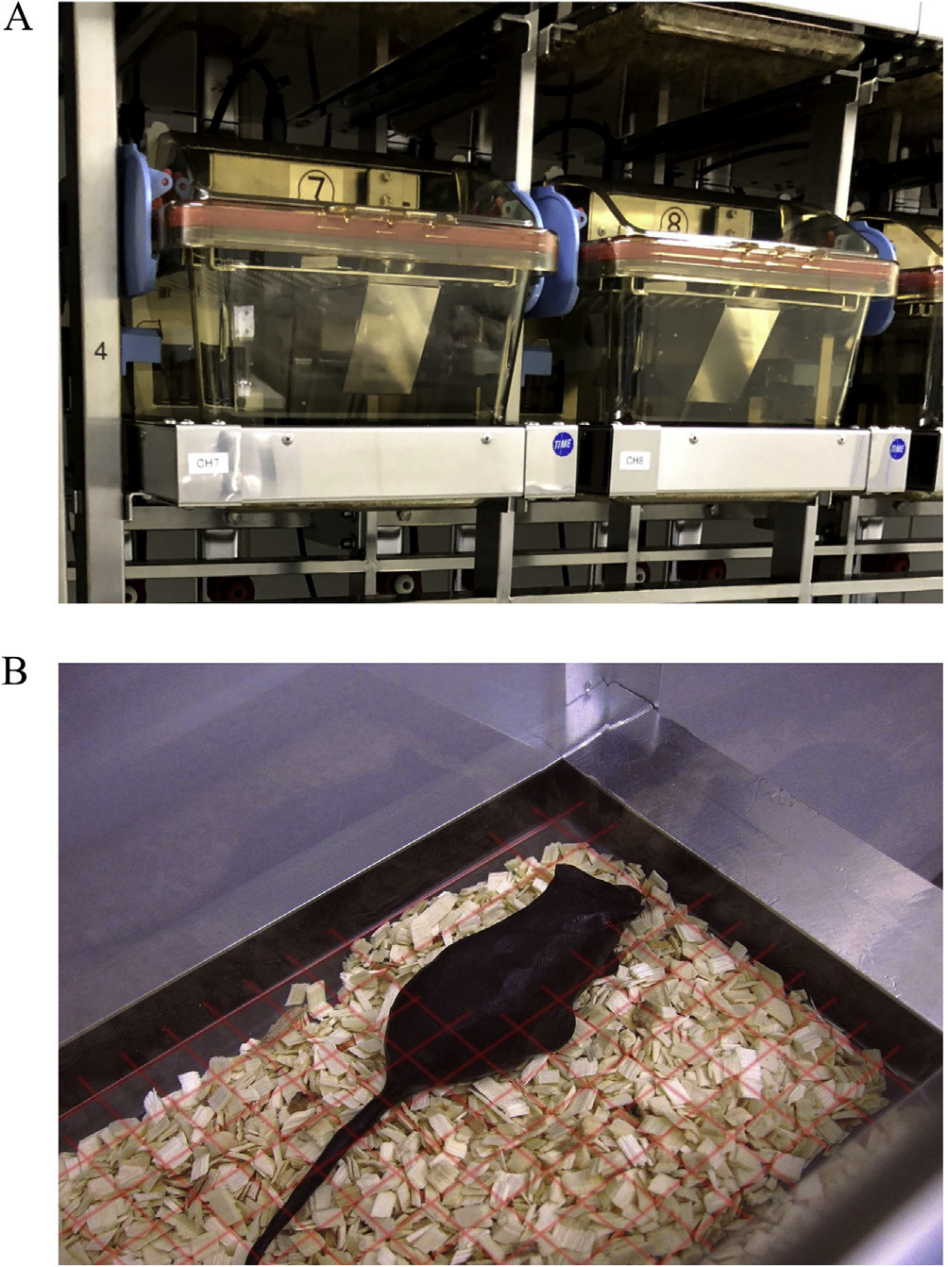
Behavioral equipment combined with sealed positive-pressure cage. Behavioral variables (activity counts and intake of water and food) of GF and SPF mice in the ISOcage P were recorded automatically by the behavioral monitoring cage (A) and activity detection system (B).

The mice were moved into the ISOcage P with the behavioral (activity, water and food intake) monitoring system (ET0225; O’HARA & CO. LTD., Tokyo, Japan) (Fig. 1A) at least 48 h prior to testing (day 0). In this cage system, infrared beams are emitted along the x- and y-axes of the cage (Fig. 1B). When the animal moves through a beam, the beam path is broken, and the number of intercepted beam is detected. The animal's activity is determined based on movement detected by the infrared beams. After habituation, we recorded alterations in mouse behavior, including activity and the intake of water and food, for 5 days (days 2–6) using the system. After behavioral data had been collected, the mice were moved back into Sentry SPP isolation cages. Fresh fecal samples, soiled bedding, swabs from Sentry SPP isolation cages, and RO water from the drinking bottles were collected 1 week before and 1 month after behavioral observations.

### 2.3 Gross morphological experiments

Fresh fecal samples, soiled bedding, swabs from the Sentry SPP isolation cages, and RO water from drinking bottles were collected, and body weight was assessed. The mice were anesthetized with sodium pentobarbital (100 mg/kg ip) and euthanized, and their adipose tissue weight (including subcutaneous and visceral fat) was examined.

### 2.4 Data analysis

Data are presented as means ± standard errors of the mean. All statistical analyses were performed using MATLAB (The Mathworks, Inc., Natick, MA, USA). Data were analyzed using analysis of variance (ANOVA), followed by the Tukey–Kramer post hoc test for multiple comparisons between groups, when appropriate.

## 3 Results

### 3.1 Microbiological tests

Test results confirmed the maintenance of the statuses of 59 GF and 53 SPF mice in the entirety of each cage before and after the behavioral experiments and before the gross morphological experiments (Tables 1 and 2).

**Table 1:** The results of microbiological tests for GF status.

Aerobic/Anaerobic	Culture media	Culture temperature	Sample	before gross morphological experiments	before behavioral experiments	after behavioral experiments
Anaerobic	Cooked meat broth	37 °C	Fece	0/20	0/39	0/39
			Bedding	0/20	0/39	0/39
			Swab	0/20	0/39	0/39
			RO water in the bottle	0/20	0/39	0/39
		Room Temperature	Fece	0/20	0/39	0/39
			Bedding	0/20	0/39	0/39
			Swab	0/20	0/39	0/39
			RO water in the bottle	0/20	0/39	0/39
	Thioglycollate medium	37 °C	Fece	0/20	0/39	0/39
			Bedding	0/20	0/39	0/39
			Swab	0/20	0/39	0/39
			RO water in the bottle	0/20	0/39	0/39
		Room Temperature	Fece	0/20	0/39	0/39
			Bedding	0/20	0/39	0/39
			Swab	0/20	0/39	0/39
			RO water in the bottle	0/20	0/39	0/39
Aerobic	Heart infusion broth	37 °C	Fece	0/20	0/39	0/39
			Bedding	0/20	0/39	0/39
			Swab	0/20	0/39	0/39
			RO water in the bottle	0/20	0/39	0/39
		Room Temperature	Fece	0/20	0/39	0/39
			Bedding	0/20	0/39	0/39
			Swab	0/20	0/39	0/39
			RO water in the bottle	0/20	0/39	0/39
	Potate dextrose broth	Room Temperature	Fece	0/20	0/39	0/39
			Bedding	0/20	0/39	0/39
			Swab	0/20	0/39	0/39
			RO water in the bottle	0/20	0/39	0/39

**Table 2:** The results of microbiological tests for GF status.

Microorganism	before gross morphological experiments	before behavioral experiments	after behavioral experiments
Mouse hepatitis virus	0/18	0/35	0/35
Sendai virus	0/18	0/35	0/35
Ectromelia virus	0/18	0/35	0/35
Lymphocytic choriomeningitis virus	0/18	0/35	0/35
Mouse rotavirus	0/18	0/35	0/35
Mouse parvovirus	0/18	0/35	0/35
Mouse encephalomyelitis virus	0/18	0/35	0/35
Pneumonia virus of mice	0/18	0/35	0/35
Mouse adenovirus, reovirus type 3	0/18	0/35	0/35
Lactate dehydrogenase elevating virus	0/18	0/35	0/35
*Mycoplasma pulmonis*	0/18	0/35	0/35
*Salmonella typhimurium*	0/18	0/35	0/35
*Clostridium piliforme*	0/18	0/35	0/35
*Pasteurella pneumotropica*	0/18	0/35	0/35
*Cilia-associated respiratory bacillus*	0/18	0/35	0/35
*Escherichia coli O115a*	0/18	0/35	0/35
*Helicobacter hepaticus*	0/18	0/35	0/35
*Pseudomonas aeruginosa*	0/18	0/35	0/35
*Pneumocystis carinii*	0/18	0/35	0/35
*Syphacia obvelata*	0/18	0/35	0/35
*Aspiculuris tetraptera*	0/18	0/35	0/35

### 3.2 Behavioral experiments

**Fig. 2:**
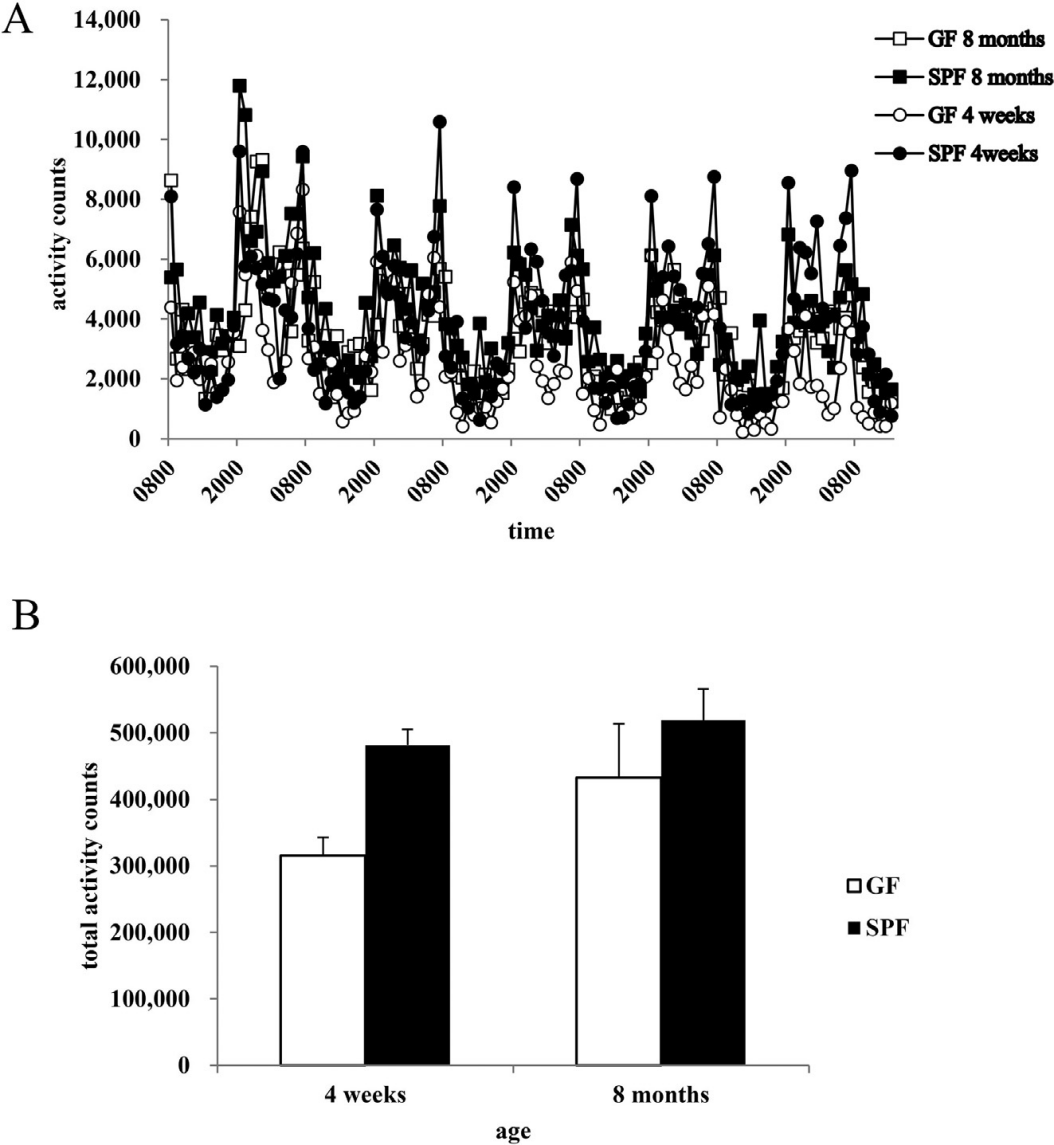
Activity of GF and SPF mice. Time-specific (A) and total (B) activity counts of GF and SPF mice at the ages of 4 weeks and 8 months during the test period (days 2–6).

The activity counts for mice of each strain were plotted for the 5 recording days (days 2–6; Fig. 2A). Both strains of mouse displayed increases in activity after the lights had been turned off and surges of activity when the lights were turned on. Fig. 2B shows the total activity counts over 5 days (days 2–6). A 2 (strain) × 2 (age) ANOVA revealed significant differences in activity between groups [strain × age interaction: *F*(1,34) = 0.74, *P* > 0.05; strain effect: *F*(1,34) = 7.47, *P* < 0.01; age effect: *F*(1,34) = 2.81, *P* > 0.05]. The activity of GF mice did not differ from that of age-matched SPF mice at the age of 4 weeks [*P* = 0.060 (Tukey–Kramer test)] or 8 months [*P* = 0.577 (Tukey–Kramer test)].

**Fig. 3:**
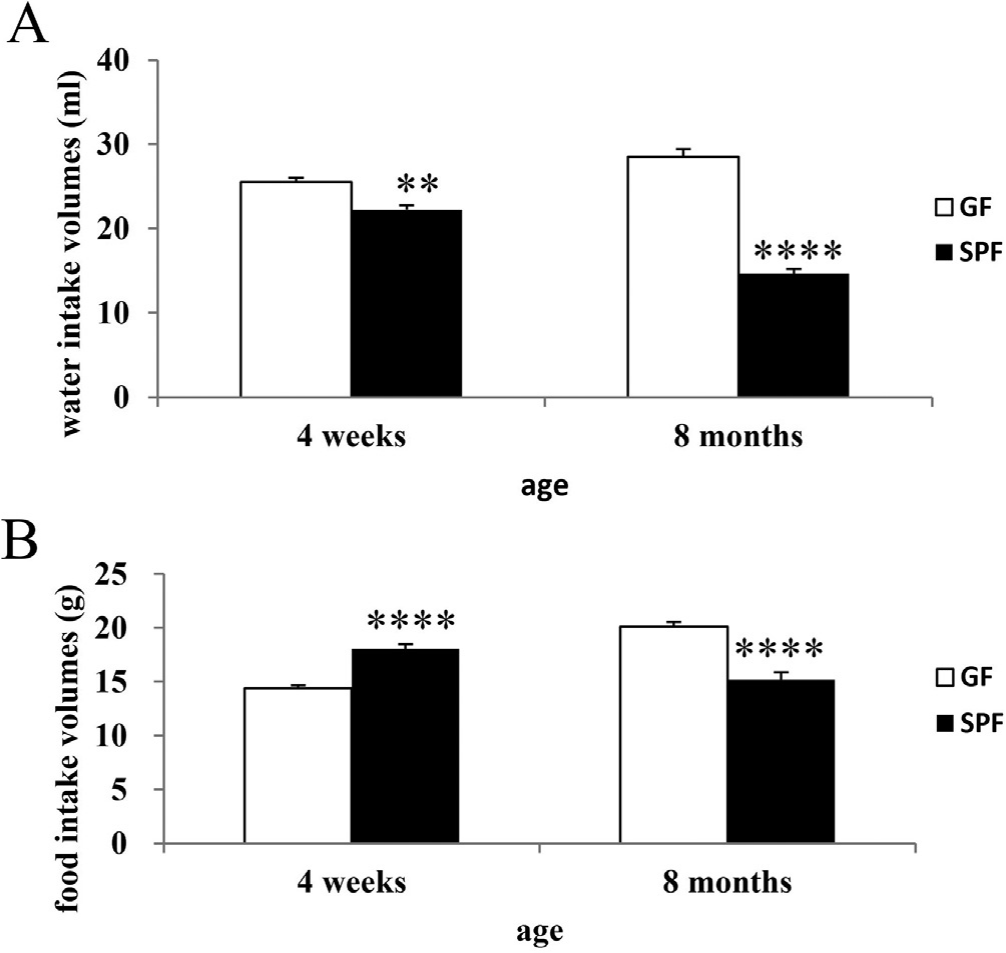
Water and food intakes of GF and SPF mice. Water intake volumes (A) and food intake weights (B) of GF and SPF mice at the ages of 4 weeks and 8 months during the test period (days 2–6). ****P < 0.001, **P < 0.01, compared with the appropriate control (Tukey–Kramer test).

Fig. 3A shows the total water intakes over the 5 days (days 2–6). A 2 (strain) × 2 (age) ANOVA revealed significant differences in water intake between groups [strain × age interaction: *F*(1,34) = 69.06, *P* < 0.001; strain effect: *F*(1,34) = 180.48, *P* < 0.001; age effect: *F*(1,34) = 48.47, *P* < 0.01]. The water intakes of GF mice were significantly higher than those of age-matched SPF mice at the ages of 4 weeks [*P* < 0.01 (Tukey–Kramer test)] and 8 months [*P* < 0.001 (Tukey–Kramer test)].

Fig. 3B shows the total food intakes over the 5 days (days 2–6). A 2 (strain) × 2 (age) ANOVA revealed significant differences in food intake between groups [strain × age interaction: *F*(1,32) = 77.67, *P* < 0.001; strain effect: *F*(1,32) = 1.79, *P* > 0.05; age effect: *F*(1,32) = 8.31, *P* < 0.01]. The food intakes of GF mice were significantly lower than those of age-matched SPF mice at 4 weeks of age [*P* < 0.001 (Tukey–Kramer test)] and significantly higher than those of SPF mice at 8 months of age [*P* < 0.001 (Tukey–Kramer test)].

### 3.3 Gross morphological experiments

**Fig. 4:**
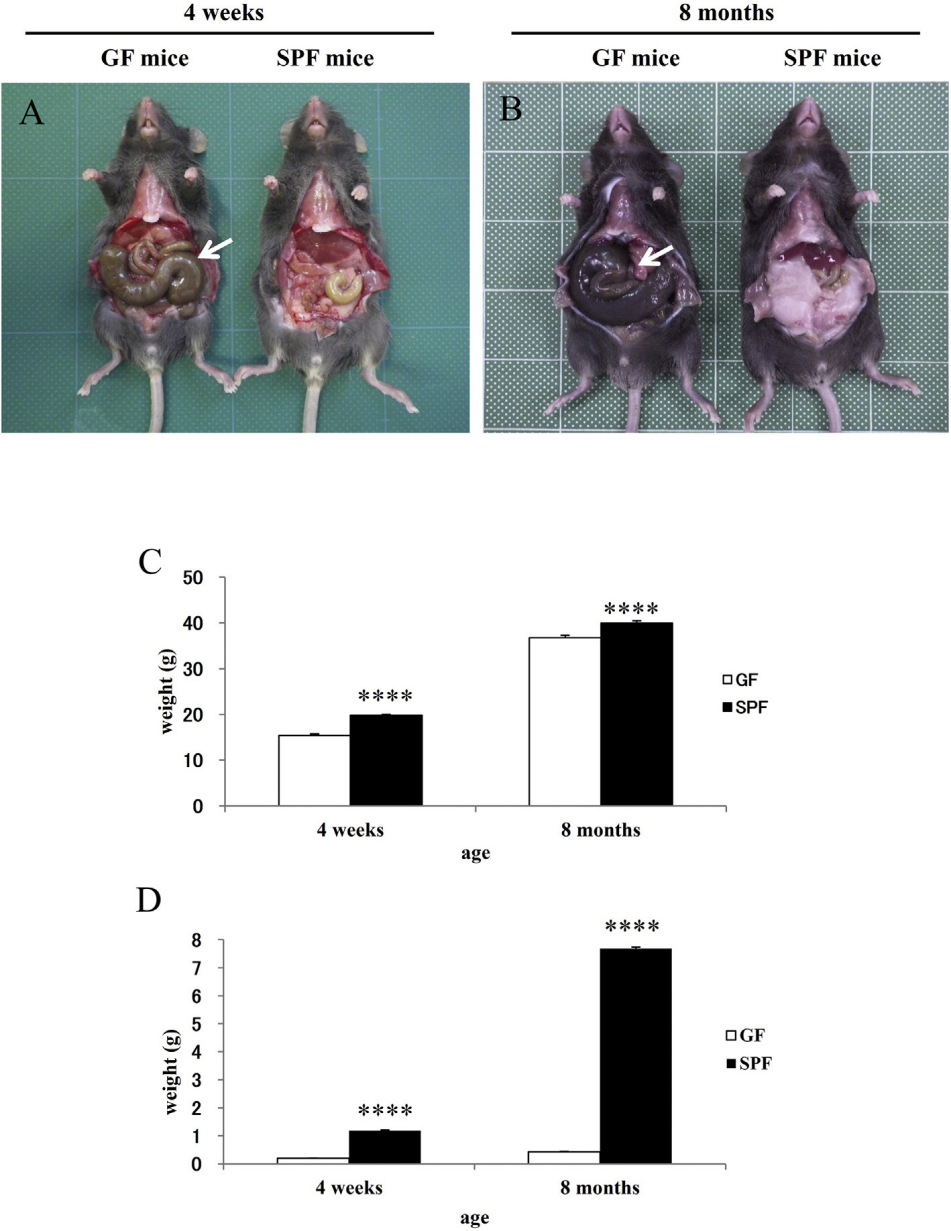
Representative photographs of the intestines, and the body weights and adipose tissue weights, of GF and SPF mice. The ceca are indicated by white arrows in GF (left) and SPF (right) mice at 4 weeks (A) and 8 months (B). Body (C) and adipose tissue (D) weights of GF and SPF mice at the ages of 4 weeks and 8 months during the test period (days 2–6). ****P < 0.001, compared with appropriate control (Tukey–Kramer test).

No gross abnormality was observed in the brain, lung, heart, liver, pancreas, or kidney of any 4-week-old or 8-month-old GF or SPF mouse (data not shown). Cecal enlargement (indicated by white arrows) was the most striking anomaly in GF mice at ages 4 weeks (Fig. 4A) and 8 months (Fig. 4B).

The body weights of the 4-week-old GF, 4-week-old SPF, 8-month-old GF, and 8-month-old SPF mice are shown in Fig. 4C. A 2 (strain) × 2 (age) ANOVA revealed significant differences in body weight between groups [strain × age interaction: *F*(1,34) = 1.91, *P* > 0.05; strain effect: *F*(1,34) = 76.65, *P* < 0.001; age effect: *F*(1,34) = 2275.19, *P* < 0.001]. The body weights of GF mice were significantly lower than those of age-matched SPF mice at the ages of 4 weeks [*P* < 0.001 (Tukey–Kramer test)] and 8 months [*P* < 0.001 (Tukey–Kramer test)].

The adipose tissue weights, including subcutaneous and visceral fat, of the 4-week-old GF, 4-week-old SPF, 8-month-old GF, and 8-month-old SPF mice are shown in Fig. 4D. A 2 (strain) × 2 (age) ANOVA revealed significant differences in adipose tissue weight between groups [strain × age interaction: *F*(1,34) = 11,234.1, *P* < 0.001; strain effect: *F*(1,34) = 19,460.7, *P* < 0.001; age effect: *F*(1,34) = 12,998.9, *P* < 0.001]. The adipose tissue weights of the GF mice were significantly lower than those of age-matched SPF mice at the ages of 4 weeks [*P* < 0.001 (Tukey–Kramer test)] and 8 months [*P* < 0.001 (Tukey–Kramer test)].

## 4 Discussion

If an error in isolator handling occurs, all cages in the isolator are at risk of contamination, although GF mice are usually reared in flexible-film isolators. When research necessitates the parallel inoculation of GF mice with a variety of microorganisms, the isolator system mandates that each group be housed individually in a different isolator to prevent cross-contamination between study groups, which is space intensive and limits research throughput. To overcome these disadvantages, a new individually SPP cage that promises more standardized cage conditions and reduced operational costs was developed and used in previous studies [11, 12, 13]. In this study, we also maintained GF and SPF mice in the same rack in individual Sentry and ISOcage P SPP cages. However, isolators are also disadvantageous because they render the observation of mouse behavior, such as activity and the intake of water and food, under GF status difficult. To ameliorate this issue, we combined a novel behavioral testing system featuring the automated collection of data on GF mice, such as activity counts and the intake of water and food, with SPP isolation cages in this study.

We first measured and compared the levels of activity and the intakes of water and food between the two strains at 4 weeks and 8 months of age. The circadian rhythms of the GF and SPF mice were similar. Although the GF mice exhibited a tendency for decreased activity compared with age-matched SPF mice, the activity of GF mice did not differ significantly from that of age-matched SPF mice at the age of 4 weeks or 8 months.

The GF mice exhibited increased water intakes compared with age-matched SPF mice.

The 4-week-old GF mice exhibited decreased food intake, whereas the 8-month-old GF mice exhibited increased food intake, compared with age-matched SPF mice. The SPF mice exhibited decreases in water intake that were age dependent, whereas the GF mice exhibited increases that were age dependent. The GF mice had lower body weights than age-matched SPF mice, and both strains exhibited increases in body weight that were age dependent. Conversely, the GF mice had much lower adipose tissue weights than age-matched SPF mice. The SPF mice exhibited increases that were age dependent, whereas the GF mice had little gain in their adipose tissue weights. In a previous study [14], mice with depletion of microbiota by antibiotic treatment demonstrated a decrease in adipose tissue weight and increased food intake. These findings are similar to the data we obtained using GF mice in this study. Antibiotic treatment in mice leads to the browning of adipose tissue in subcutaneous and visceral depots [14]. Although this study did not involve examination of the relationships among body weight, adipose tissue weight, activity, water intake, and food intake, GF mice would be useful models for the examination of related metabolic functions.

Enlargement of the cecum was observed in GF mice compared with SPF mice. Cecal enlargement in GF mice is thought to be caused by retention of water that is attracted to the accumulated mucus and undigested fibers in the cecal lumen [15, 16, 17]. These results also demonstrate that our current system allowed us to examine the behaviors of these mice without bacterial contamination.

Humans harbor complex microbial communities, with the vast majority of the microbial population residing in the distal gut. Gut microbes perform key functions for human health, including energy extraction, biosynthesis of vitamins, protection against pathogen overgrowth, and training of the immune system [18]. Microbial colonization of the gut occurs during birth, is highly dynamic through infancy, and resembles the adult structure by about 3 years of age [19]. Thereafter, the composition of the microbiome in an individual remains generally stable [20], although substantial interpersonal variation exists, particularly in elderly individuals [21]. Alterations in the composition of this complex ecosystem have been associated with the development of a variety of gastrointestinal and metabolic diseases, including inflammatory bowel disease, obesity, diabetes, and insulin resistance [22, 23]. More recently, the influence of the gut microbiota on central nervous system function – often referred to as the gut–brain axis – has received significant attention, and alterations in the gut microbiome have been associated with neurological conditions, including autism spectrum disorder, multiple sclerosis, Parkinson's disease, and Alzheimer's disease [24, 25, 26, 27, 28].

In this study, GF mice were housed in an SPP cage and their behaviors were examined in a combined behavioral equipment–SPP cage system. We automatically recorded alterations in activity and the intake of water and food in GF mice over time. To our knowledge, this report is first to specifically analyze these behaviors in GF mice. With increased use of GF or gnotobiotic mice as models, a system that minimizes unwanted contamination while allowing researchers easier access for the measurement of behavioral characteristics is needed. We believe that our platform has many advantages that will enable it to serve as an ideal system for the examination of metabolic functions and host–microbe interactions.

## Declarations

### Author contribution statement

Kimie Niimi: Performed the experiments; Analyzed and interpreted the data; Contributed reagents, materials, analysis tools or data.

Eiki Takahashi: Conceived and designed the experiments; Analyzed and interpreted the data; Wrote the paper.

### Funding statement

This work was supported by the cross-disciplinary project of the Integrated Life Science Research to Challenge Super Aging Society at RIKEN.

### Competing interest statement

The authors declare no conflict of interest.

### Additional information

No additional information is available for this paper.
